# MAGNet: A Camouflaged Object Detection Network Simulating the Observation Effect of a Magnifier

**DOI:** 10.3390/e24121804

**Published:** 2022-12-09

**Authors:** Xinhao Jiang, Wei Cai, Zhili Zhang, Bo Jiang, Zhiyong Yang, Xin Wang

**Affiliations:** Xi’an Research Institute of High Technology, Xi’an 710064, China

**Keywords:** camouflaged object detection, image segmentation, deep learning, human visual system, computer vision

## Abstract

In recent years, protecting important objects by simulating animal camouflage has been widely employed in many fields. Therefore, camouflaged object detection (COD) technology has emerged. COD is more difficult to achieve than traditional object detection techniques due to the high degree of fusion of objects camouflaged with the background. In this paper, we strive to more accurately and efficiently identify camouflaged objects. Inspired by the use of magnifiers to search for hidden objects in pictures, we propose a COD network that simulates the observation effect of a magnifier called the MAGnifier Network (MAGNet). Specifically, our MAGNet contains two parallel modules: the ergodic magnification module (EMM) and the attention focus module (AFM). The EMM is designed to mimic the process of a magnifier enlarging an image, and AFM is used to simulate the observation process in which human attention is highly focused on a particular region. The two sets of output camouflaged object maps were merged to simulate the observation of an object by a magnifier. In addition, a weighted key point area perception loss function, which is more applicable to COD, was designed based on two modules to give greater attention to the camouflaged object. Extensive experiments demonstrate that compared with 19 cutting-edge detection models, MAGNet can achieve the best comprehensive effect on eight evaluation metrics in the public COD dataset. Additionally, compared to other COD methods, MAGNet has lower computational complexity and faster segmentation. We also validated the model’s generalization ability on a military camouflaged object dataset constructed in-house. Finally, we experimentally explored some extended applications of COD.

## 1. Introduction

In nature, animals evolve according to the principle of survival of the fittest. They may be able to camouflage their shape or retain shape characteristics similar to those of their habitat to avoid being hunted by predators or to ambush prey better [1]. Currently, following the recent progress in various fields of science and technology, camouflage technology that simulates animal camouflage, such as camouflage clothing and nets [2], has been widely used in modern warfare.

In addition to its military application, camouflaged object detection (COD) can be applied in industrial detection (e.g., equipment defect detection [3]), medical diagnoses (e.g., testing whether lungs are infected with pneumonia [4,5]), monitoring and protection (e.g., suspicious person or unmanned aerial vehicle intrusion detection [6,7]), and unmanned driving (e.g., road obstacle detection [8]).

The task of COD is to detect objects that have similar patterns (e.g., color and texture) to their surroundings. However, studies on COD are lacking. For example, in military fields, military camouflaged objects are often identified by means of infrared-, polarization-, and hyperspectral-based imaging and other technologies [9,10,11]. However, the challenge of accurately segmenting camouflaged objects in the visible light band has been largely neglected in scientific research. Despite this, television guidance is still widely used by most countries because of its low cost and better visualization. The common method of countering television guidance is to camouflage the object, so the study of COD for visible images is greatly significant in military applications.

Before the rapid development of deep learning, researchers commonly used traditional digital image processing methods, such as spectral transforms [12], sparse matrices [13], and human vision systems [14]. However, the traditional methods achieve image segmentation by some artificially set rules. Primarily, scholars theoretically argue for the rationality of the rule and then experimentally verify its effectiveness. Nevertheless, actual segmentation scenes are often more complex than validation scenes, and predefined rules cannot be flexibly adjusted according to image features, which leads to less-than-ideal results from traditional methods (Some of the experimental results are published at https://github.com/jiangxinhao2020/Magnet_eval (accessed on 1 December 2022)). Now, with the development of deep learning technology, some scholars have applied segmentation to the detection of camouflaged objects, providing new ideas for the detection of camouflaged objects in visible wavelengths [15]. However, existing COD models based on deep learning are often complex in terms of design principles and network structure in the pursuit of higher accuracy rates, which will make the computational complexity and number of parameters of the model large.

**Figure 1:**
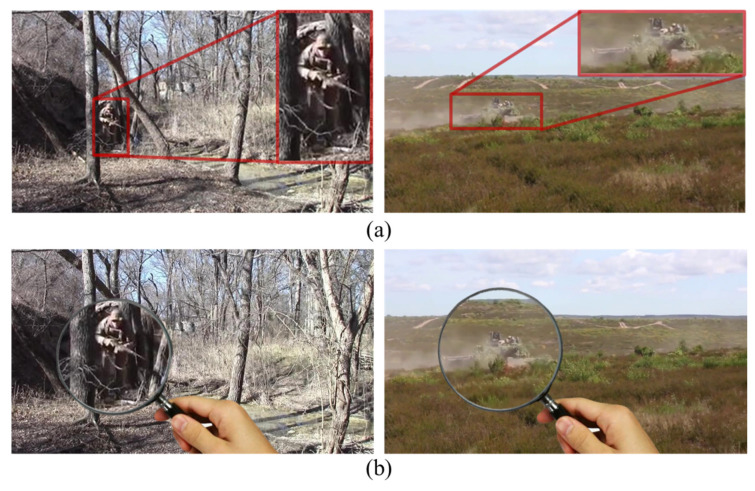
Schematic diagram of the observation of a camouflaged soldier and tank with a magnifier. (**a**) Camouflaged objects; (**b**) Observe camouflage objects with a magnifier.

The originality of this study is that, instead of rigidly solving the problem from the perspective of deep learning, we took our inspiration from life observations and designed a segmentation network suitable for camouflaged objects by simulating the magnifying glass observation effect on a target. This is called the MAGnifier Network (MAGNet). MAGNet differs from other COD methods in that it has a clearer structure and can achieve a better segmentation performance with lower computational complexity. Figure 1 is a schematic diagram demonstrating a search for camouflaged military objects based on observation with a magnifier. With the influence of the camouflage coating, external camouflage materials, smoke barriers, and ground object shielding, the soldier and tank in Figure 1a achieve near-perfect integration with the background. However, Figure 1b shows that the camouflaged objects in the picture can be simply and effectively observed with a magnifier. Firstly, the magnifier visually enlarges the observation area, and we can then observe edge information and key parts of camouflaged objects in the enlarged area. Therefore, we can focus on the key points to accurately identify camouflaged objects in the region.

In summary, the major contributions of this paper are threefold:

1. We apply the concept of observation with a magnifier to the COD problem and propose a novel camouflaged object segmentation network called MAGNet with a clear structure. MAGNet can achieve higher segmentation accuracy with lower computational complexity.
2. We design a parallel structure with the ergodic magnification module (EMM) and attention focus module (AFM) to simulate the magnifier functions. We propose a weighted key point area perception loss function to improve the focus of the camouflaged object, thus improving segmentation performance.
3. We perform extensive experiments using public COD benchmark datasets and a camouflaged military object dataset constructed in-house. MAGNet has the best comprehensive effect in eight evaluation metrics in comparison with 19 cutting-edge detection models, and it can enable real-time segmentation. Finally, we experimentally explore several potential applications of camouflaged object segmentation.

This paper is organized as follows. Similar previous research is introduced in Section 2. Section 3 provides detailed descriptions of our MAGNet and the associated modules. Section 4 presents comparative experiments and quantitative and qualitative analyses of the experimental results. Finally, Section 5 concludes the paper.

## 2. Related Research

### 2.1. Semantic Segmentation Based on Deep Learning

In recent years, scene understanding technologies for use in autonomous driving [16], virtual reality [17], and augmented reality [18] have rapidly developed. As the basic scene understanding task, semantic segmentation technology based on pixel-by-pixel classification has been widely studied [19,20,21]. Many semantic segmentation methods based on deep learning have been proposed [22,23,24,25]. Currently, there are four main types of networks: fully convolutional networks (FCNs) [26], convolutional neural networks (CNNs) [27], recurrent neural networks (RNNs) [28], and generative adversarial networks (GANs) [29].

### 2.2. Salient Object Detection Based on Deep Learning

In contrast to camouflaged objects, salient objects are the most noticeable objects in an image. The research of salient object detection (SOD) can promote image understanding [30], stereo matching [31,32], and medical disease detection [33,34,35]. In recent years, salient object detection based on deep learning has been improved by multi-scale feature fusion [36], attention mechanisms [37], and edge information [38]. Research on SOD can provide insights into COD in terms of design principles.

### 2.3. Camouflaged Object Detection Based on Deep Learning

**Figure 2:**
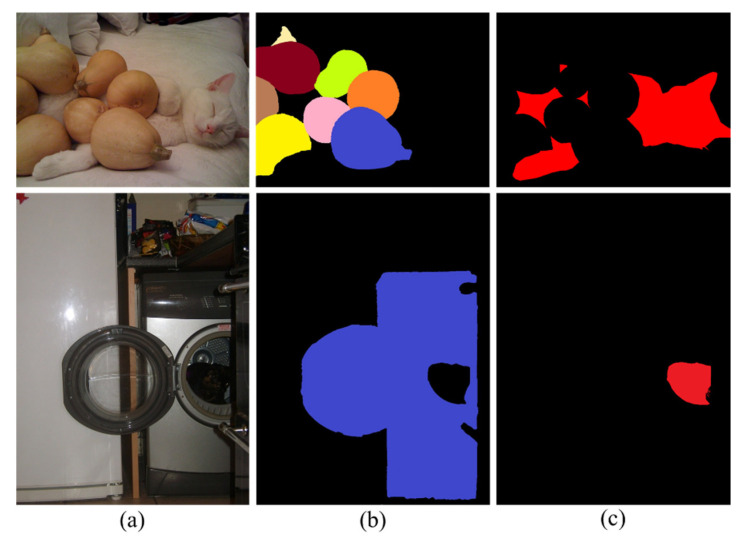
The difference between camouflage objects and salient objects. (**a**) Image; (**b**) Salient object; (**c**) Camouflaged object.

Figure 2 shows the difference between a camouflaged object and a salient object. As can be seen, COD is more difficult than SOD. It should be noted that scholars commonly use the terms “camouflage target segmentation” and “camouflage object detection” interchangeably; therefore, this paper continues to use the term COD. The year 2020 can be regarded as the first year of research on COD based on deep learning. Fan et al. [39] constructed a complete camouflaged object dataset named COD10K and presented a corresponding camouflaged object segmentation network that promotes rapid COD development. In 2021, Mei et al. [40] simulated the predation process of animals and proposed PFNet, a camouflaged object segmentation network based on distraction mining. Lv et al. [41] proposed a joint learning network that can simultaneously localize, segment, and rank camouflaged objects and proposed a new COD dataset called NC4K. However, the design principles and network structures of the existing COD models are relatively complex. This paper presents a bionic model based on observation with a magnifier. This principle is easy to understand, and its structure is simple and efficient.

### 2.4. COD Dataset

Because of the similarity between a camouflaged object and the background, the boundary between the foreground and the background is very difficult to distinguish; therefore, the production of a camouflaged object dataset is very time-consuming [42]. Currently, three major published datasets are the most commonly used. The number of images in the CHAMELEON dataset is small, with only 76 published images collected from the internet [43]. The CAMO dataset contains 1250 images in eight categories [44]. In 2020, Fan et al. proposed the COD10K universal camouflaged object dataset, which has 78 subclasses of 10K images, and this dataset is very precise and challenging [39].

## 3. MAGNet Detection Model

**Figure 3:**
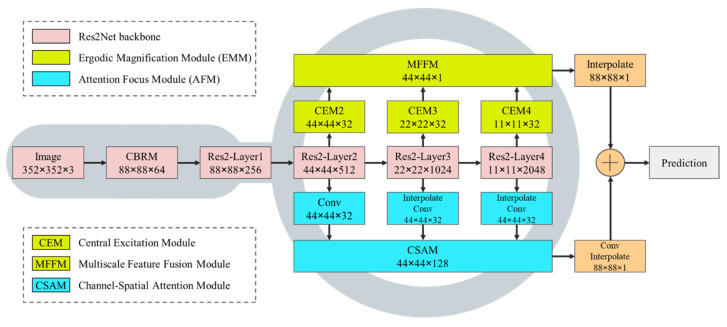
MAGNet structure.

A magnifier can help an observer quickly find a camouflaged object in an image. This is because the magnifying effect of the magnifier makes it easier for the observer to spot the center, key points, and minuscule details of the camouflaged object. Inspired by the magnifier, we applied the magnifier observation effect to the COD problem and designed the EMM and the AFM. The EMM is designed to mimic the process of a magnifier enlarging an image, mainly using the designed central excitation module to excite the center and magnify the receptive field. Additionally, AFM is used to simulate the human visual system, and its channel-spatial attention module can simulate the effect of a human focusing on observing objects in the magnifier’s field of view. Finally, we design a more applicable weighted key point area perception loss function for camouflaged object segmentation, which directs more attention to the camouflaged object in the region by weighting. The network structure of MAGNet is shown in Figure 3, which has a clear structure with two sets of branches forming a parallel structure and finally fuses two sets of feature maps to achieve the final camouflage object recognition.

### 3.1. Network Overview

We input a camouflaged object image into this network. MAGNet first extracts multi-scale feature maps through a Res2Net-50 backbone [45], and Res2Net consists of one layer of CBRM (Conv+BatchNorm+ReLU Module) and four standard Res2-Layers. Additionally, the latter three feature maps were then fed to the EMM and the AFM in parallel. Finally, the output feature maps of the two modules are fused to simulate observation with a magnifier.

### 3.2. Ergodic Magnification Module (EMM)

As shown in Figure 3, the EMM consists of two parts, i.e., the central excitation module (CEM) and the multi-scale feature fusion module (MFFM).

The CEM is used to traverse the feature maps of the different scales of output from the last three layers of the backbone to expand the receptive field and intensify the central point and key points.

The MFFM is designed to fully integrate the multi-scale feature maps after the CEM to realize the efficient utilization of high-level and low-level features.

#### 3.2.1. Central Excitation Module (CEM)

We find that when observers use a magnifier to observe an object, they observe the central area of the magnifying glass more carefully than the edge areas. With the human visual receptive field mechanism, an observer is more attracted to the center of an object. Then, we use the magnifier to traverse the whole picture until the center of the magnifier coincides with the center of the object. Several studies [46] discuss the differences between the receptive field mechanism in deep learning and the biological receptive field mechanism through a large number of experiments and points out that the value of the pixel in the center of the receptive field responds more to the output feature map than the pixel at the edges. This inspired us to design a receptive field mechanism not only to expand the receptive field but also to motivate key points.

**Figure 4:**
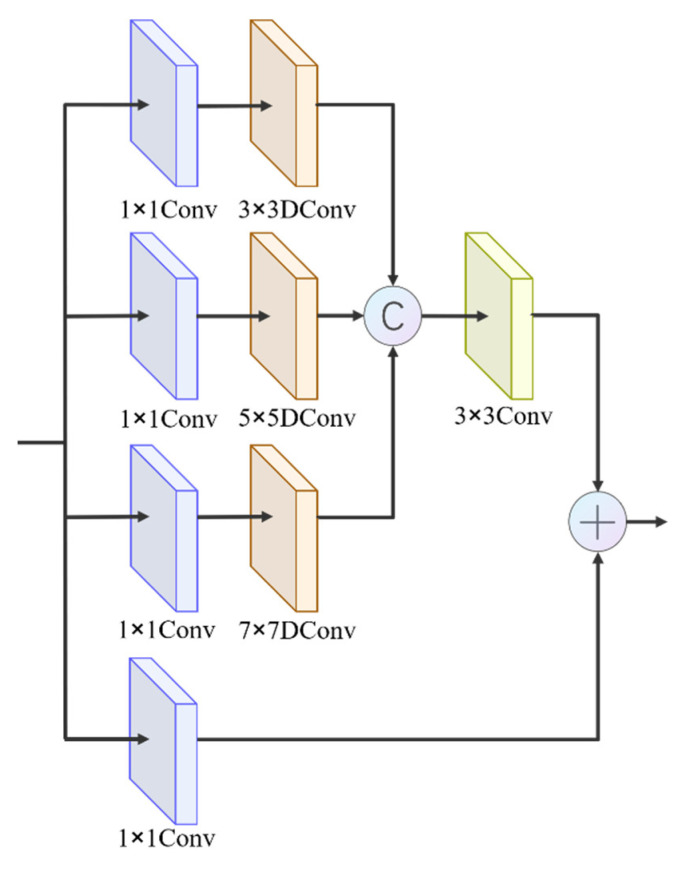
The structure of the CEM.

To simulate the visual magnification and central excitation of the magnifier, we design a simple and efficient CEM, as shown in Figure 4. The realization of the above functions mainly depends on dilated convolution (DConv) with different sizes of convolution kernels [47].

Specifically, the CEM consists of four branches, and the input feature maps are simultaneously fed into all four branches. The four branches first use a 1 × 1 convolution to change the number of output channels. Then, to achieve efficient multi-scale visual amplification, three of the branches use 3 × 3, 5 × 5, and 7 × 7 DConvs with an expansion factor of 2. After the three sets of output feature maps were connected, a 3 × 3 convolutional layer was used for fusion between channels. The fourth branch is the residual connection module, which aims to retain part of the original features to reduce the feature loss due to convolution. The two sets of features are connected to obtain a centrally excited feature map. The multi-scale centrally excited feature maps obtained from the last three layers of backbone input to the CEM have the same number of channels to ensure a balanced utilization of information at each scale.

**Figure 5:**
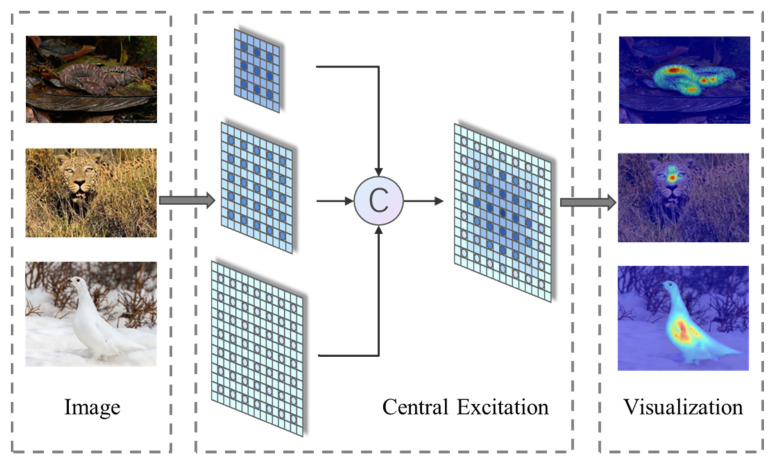
Schematic diagram of the central excitation effect of CEM.

The connection of three sets of DConvs can increase the importance of the central features while increasing the receptive field, thus achieving a central excitation of the input. The visualization of the feature map output from the CEM is shown on the right in Figure 5.

#### 3.2.2. Multi-Scale Feature Fusion Module (MFFM)

**Figure 6:**
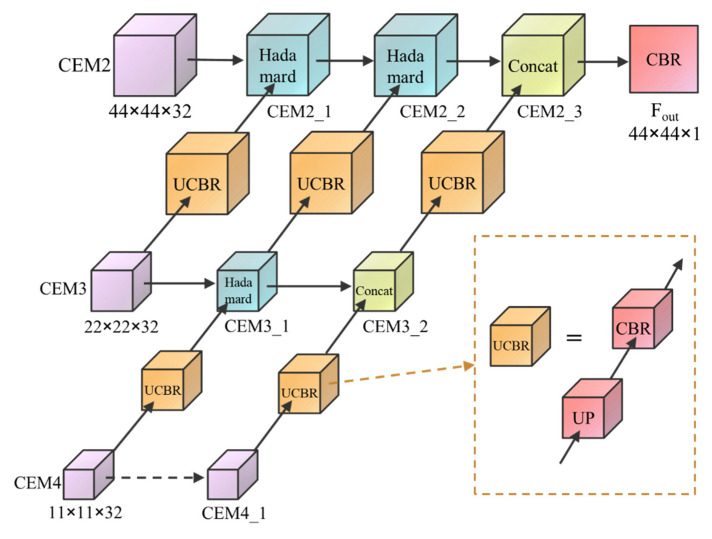
Structure of the MFFM module. (UP: upsample, CBR: Conv+BatchNorm+ReLU).

The function of the MFFM is to fully integrate the feature maps after central excitation of different scales, thereby outputting a camouflaged object map that contains abundant high- and low-level features. The MFFM structure diagram is shown in Figure 6. The small-scale excitation feature map transmits the feature information to the large-scale feature map through continuous upsampling and fusion and then generates an output feature map with a size of 44 × 44 × 1.

The front-end fusion method of the module adopts the Hadamard product (⊙). The Hadamard product calculation method is a pixel-by-pixel multiplication, which can better achieve feature crossover, eliminating the difference between the two groups of features and improving the feature fusion capabilities.

The back end of the module is fused by adding the channels, which can fuse the features of each layer to increase the feature dimension but does not increase the internal feature information, making full use of the semantic information of the high-level and low-level features.

The module output map is denoted as F_out_. Algorithm 1 is the pseudocode of the MFFM:
**Algorithm 1:** MFFM Algorithm**Input:** CEM2, CEM3, CEM4. 
   CEM4_1 = CEM4   CEM3_1 = CBR (UP (CEM4))⊙CEM3   CEM3_2 = Concat (CEM3_1, CBR (UP (CEM4_1)))   CEM2_1 = CBR (UP (CEM3))⊙CEM2   CEM2_2 = CBR (UP (CEM3_1))⊙CEM2_1   CEM2_3 = Concat (CEM2_2, CBR (UP (CEM3_2)))   F_out_ = CBR (CEM2_3)**Output:** F_out_.

### 3.3. Attention Focus Module (AFM)

AFM has two steps. First, through upsampling and convolution operations, the three sets of feature maps output by the backbone are processed into feature maps of the same size with the same number of channels. Then, the maps are input into the channel-spatial attention module (CSAM) to simulate the effect of human attention focused on observing objects in the magnifier field of view.

#### Channel-Spatial Attention Module (CSAM)

**Figure 7:**
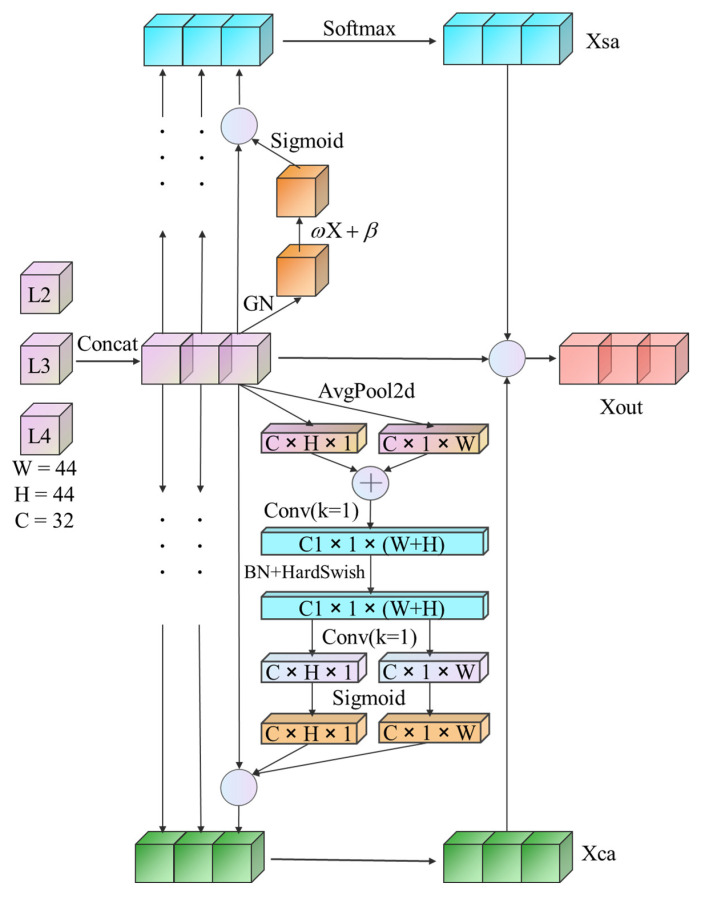
The structure of the CSAM. (H refers to the Hadamard product, L2, L3, and L4 refer to Res2-Layer2, Res2-Layer3, and Res2-Layer4, respectively).

Attention mechanisms in deep learning can simulate the human visual attention mechanism, where the goal is to obtain more important information [46]. Attention mechanisms are divided into two types: spatial attention mechanisms and channel attention mechanisms. A spatial attention mechanism module can extract the most important regional features in the spatial domain and retain locally important information by spatial transformation. A channel attention mechanism module can assign different weights according to the importance of each channel so that the model focuses more on channels with more critical information [48]. The two methods have advantages and disadvantages, and the CSAM that we propose is a parallel fusion mechanism of spatial attention and channel attention, as shown in Figure 7.

As illustrated in Figure 7, the CSAM is implemented in four steps. Algorithm 2 is the pseudocode of the CSAM:
**Algorithm 2:** CSAM Algorithm**Input:** L2, L3, L4.
   **# 1. Feature Maps Concat**   X_original = Concat(L2, L3, L4)   For i = 2, 3, 4: 
   **# 2. Spatial Attention**   xsa_i = SAmodule (Li)**   # 3. Channel Attention**   xca_i = CAmodule(Li)   Xsa = Concat (xsa_3, xsa_4, xsa_5)   Xsa = Softmax (Xsa)   Xca = Concat (xca_3, xca_4, xca_5)   **# 4. Fusion Attention Maps**   Xout = X_original ⊙ Xca ⊙ Xsa**Output:** Xout.

**Feature Maps Concat:** Superimposing the feature maps of the same size with the same number of channels in the latter three layers of the backbone after processing can achieve the average utilization of feature maps of each scale and fully fuse the semantic information of high- and low-level features. Therefore, the feature maps of the three different layers were input into the channel attention and spatial attention mechanism branches to generate a channel attention map and a spatial attention map, respectively.

**Channel Attention:** The squeeze-and-excitation (SE) module is the most commonly used method of channel attention [49]. It can extract important features by assigning weights to each channel but does not learn the importance of location information. Therefore, we embedded the coordinate attention (CA) module [50], which can fully perceive position information, into CSAM. The CA module first aggregates features near key points in the image into a pair of key point direction-aware feature maps $K_{H}(C,H,1)$ $K_{W}(C,1,W)$ with different orientations using two 2D-average-pooling operations in the horizontal and vertical dimensions.

(1) $$\begin{array}{cc} K_{W}(c,1,w)=\frac{1}{H}\sum_{0\leq i<H}F_{input}(c,i,w), & 0\leq c<C,0\leq w<W \end{array}$$

(2) $$\begin{array}{cc} K_{H}(c,h,1)=\frac{1}{W}\sum_{0\leq j<W}F_{input}(c,h,j), & 0\leq c<C,0\leq h<H \end{array}$$

where $F_{input}$ denotes the input feature maps, and the two direction-aware feature maps are fused by cascade and convolution operations, yielding the following:

(3) $$F(C1,1,W+H)=\xi (Conv_{C1}(〚K(C,H,1),K(C,1,W)〛))$$

where $〚\cdot ,\cdot 〛$ denotes the concatenation operation along the spatial dimension, $Conv_{C1}(\cdot )$ denotes the 1 × 1 convolution with C1 convolution kernels, and ξ(⋅) denotes BatchNorm and HardSwish operations on feature maps. The fused feature maps were sliced and encoded into two attention maps storing location information.

(4) $$\left\{F_{H}(C,H,1),F_{W}(C,1,W)\right\}=\delta (Conv_{C}(Slice[F(C1,1,W+H)]))$$

where Slice[⋅] denotes the slice operation along the spatial dimension and $Conv_{C}(\cdot )$ denotes the 1 × 1 convolution with C convolution kernels. δ(⋅) denotes the sigmoid activation function.

Finally, the new and old feature maps were multiplied pixel by pixel by a Hadamard convolution to generate a channel attention map with embedded location and direction information.

(5) $$\begin{array}{cc} F_{output}(c,i,j)=F_{input}(c,i,j)⊙F_{H}(c,i,1)⊙F_{W}(c,1,j), & 0\leq c<C,0\leq i<H,0\leq j<W \end{array}$$

**Spatial Attention:** The spatial attention mechanism is particularly important for finding special targets and can retain important local information. For the input feature map $F_{In}$, we first used GroupNorm (GN) for group normalization. The second step was to use a set of trainable parameters, weight (ω) and bias (β), to assign spatial weights to enhance the representation abilities of the feature map. The third step is to use a sigmoid function for activation and then multiply the $F_{In}$ pixel by pixel to obtain the spatial attention map $F_{SAM}$:

(6) $$F_{SAM}=F_{In}*\delta (\omega *GN(F_{In})+\beta )$$

Finally, we connect three sets of spatial attention maps and use softmax to normalize again.

**Fusion Channel and Spatial Attention Maps:** We use the Hadamard product for the fusion of attention maps, that is, the pixel-by-pixel multiplication method, which can better enhance feature information to obtain a more accurate feature map.

### 3.4. Output Prediction and Loss Function

Finally, the feature maps output by the EMM and AFM are transformed into a single-channel camouflaged object map through an upsampling operation. The two feature maps are fused by pixel-by-pixel addition.

The binary cross entropy (BCE) loss function and the intersection over union (IOU) loss function are the most common [51] functions for a large number of target segmentation algorithms. However, the BCE loss and IOU loss averages of all the pixel points cannot be applied to COD. In these images, camouflaged objects require more attention than other objects (especially salient objects) due to their indistinguishable characteristics.

Combining the designed pair of focusing and amplifying modules, we propose a weighted key point area perception loss based on the BCE loss and IOU loss ($L_{kap}^{w}$), adding the key point area perception weight to jointly obtain the loss function:

(7) $$L_{kap}^{w}=L_{wbce}(P,GT)+L_{wiou}(P,GT)$$

(8) $$L_{wbce}(P,GT)=-\frac{\sum_{i=1}^{H}\sum_{j=1}^{W}w_{ij}*L_{bce}(P,GT)}{\sum_{i=1}^{H}\sum_{j=1}^{W}w_{ij}}$$

(9) $$L_{wiou}(P,GT)=1-\frac{\sum_{i=1}^{H}\sum_{j=1}^{W}L_{i,j}^{enter}*w_{ij}}{\sum_{i=1}^{H}\sum_{j=1}^{W}L_{i,j}^{union}*w_{ij}}$$

where *P* is the prediction map, *GT* is the ground truth map, *H* and *W* are the picture length and width, respectively, and $L_{bce}(P,GT)$ is the original BCE loss function. The expression for the key point area perception weight $w_{i,j}$ is as follows:

(10) $$w_{i,j}=\left\{\begin{array}{cc} \left|\frac{\sum_{h,w}GT_{h,w}}{hw}-GT_{i,j}\right| & ,\frac{\sum_{h,w}GT_{h,w}}{hw}<\frac{1}{2}\\ 1-\left|\frac{\sum_{h,w}GT_{h,w}}{hw}-GT_{i,j}\right| & ,\frac{\sum_{h,w}GT_{h,w}}{hw}>\frac{1}{2} \end{array}\right.$$

where *h* and *w* are the sizes of the regions around the pixel points in the GT map, and $\sum_{h,w}GT_{h,w}$ denotes the sum of the values of all the pixel points within the region h×w centered on the pixel point (i,j) in the GT map. h and w are as small as possible because taking a value that is too large will affect model efficiency. However, it should not be smaller than the maximum perceptual field of 32 × 32 for a single pixel (i.e., the maximum number of downsampling multiples). Therefore, a region range of size 33 × 33 was selected in this experiment, and 33 as an odd number also avoids the case where the weight is equal to 1/2 and $GT_{i,j}$ is the value of the pixel point (x,y) in the GT map. From Equation (10), it can be seen that the key point area perception weight directs more attention to the camouflaged object regardless of the percentage of the camouflaged object in the region, thus making the model training favorable to segmenting camouflaged objects.

## 4. Experimental Results and Analysis

### 4.1. Preparation Work

In this experiment, the experimental platform system used was Windows 10, the GPU of the platform was an NVIDIA Quadro GV100, and the video memory was 32 GB. The CPU was an Intel Xeon Silver 4210. The experiment used the PyTorch deep learning development framework, and the computing platform was CUDA11.0. We used the Adam optimizer for network optimization during training, the image input size was set to 352 × 352, and the learning rate was set to 0.0001.

#### 4.1.1. Dataset Preprocessing

We evaluate the CAMO [44] and COD10K [39] datasets with relatively large data volumes. CAMO includes 1250 images, and COD10K includes 5066 camouflage images. The combined total of 6316 images is divided into a training set, validation set, and testing set according to a ratio of 6:2:2. In addition, we performed validation experiments on a military camouflaged object dataset that we constructed. The dataset contains 2700 images of camouflaged soldiers and tanks. The details of the dataset are shown in Table 1, and the division ratio is also 6:2:2.

#### 4.1.2. Evaluation Metrics

At present, there are many evaluation metrics suitable for COD, and each metric focuses on different points. Based on previous scholars’ research, we selected eight evaluation metrics. A brief introduction of the metrics is as follows: The structure measure ($S_{\alpha }$) is a structural similarity evaluation metric focusing on evaluating the structural information of the prediction map [52]. The weighted F-measure ($F_{\beta }^{w}$) is a comprehensive evaluation of the accuracy and recall rate of the prediction map [53]. The mean absolute error (MAE) is the sum of the absolute values of differences between the pixels of the prediction map and the GT map [54]. The adaptive enhanced alignment measure ($E_{\phi }^{ad}$) can evaluate the pixel-level similarity effect and obtain image-level statistics [55]. The mean Dice coefficient (meanDic) represents the percentage of correctly segmented area to true area in the GT image [56]. The mean intersection over union (meanIOU) is the ratio of the area of overlap and concatenation between the predicted and ground truth maps. The mean sensitivity (meanSen) measures the percentage of predicted correct results according to the GT image. The mean specificity (meanSpe) measures the percentage of predicted incorrect results according to the GT image. The FPS uses NVIDIA 3060 for evaluating segmentation speed.

#### 4.1.3. Comparison Methods

To prove the effectiveness of the MAGNet proposed in this paper, we compared it with 19 classical and state-of-the-art algorithms. These include generic object detection methods, MaskRCNN [57], HTC [58], Swin-S [59], and DetectoRS [60]; medical image segmentation methods, UNet++ [61], HarDNet [62], PraNet [5], SANet [25], CaraNet [63], and UACANet-L [64]; SOD methods BASNet [65], SCRN [66], F3Net [51], and GCPANet [67]; and COD methods SINet-V1 [39], Rank-Net [41], PFNet [40], SINet-V2 [68], and ZoomNet [69]. For a fair comparison of segmentation performance, all algorithms are trained, validated, and tested using the partitioned dataset discussed in Section 4.1.1, and the input sizes are set to 352 × 352. In addition, the evaluation metrics are calculated using the same set of codes. The evaluation code uses the toolboxes disclosed by PFNet [40] and SINet-V2 [68].

### 4.2. Comparison with State-of-the-Art Algorithms on Public Datasets

#### 4.2.1. Quantitative Comparison

**Figure 8:**
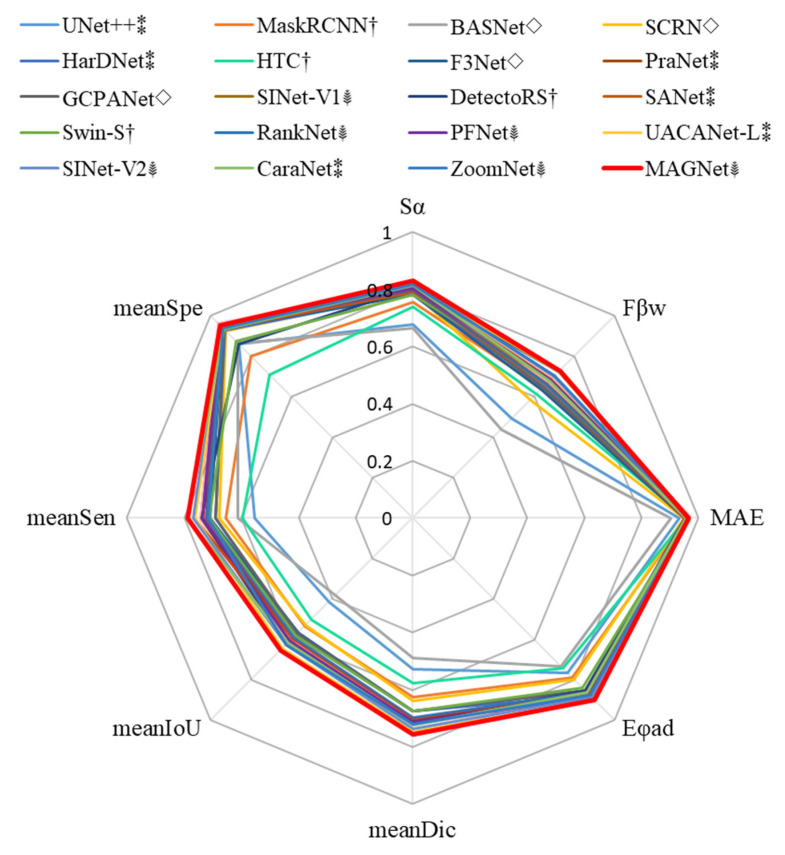
Comparison of the algorithm’s radar plot on each indicator. (For the convenience of display, set MAE = 1 − MAE; best viewed in color. †: generic object detection methods, ⁑: medical image segmentation method, ◊: saliency object detection method, ⸙: COD method.).

Table 2 comprehensively reports the quantitative results of MAGNet and the latest algorithms on the combined dataset. Figure 8 is a radar plot of eight indicators. As seen from the table, MAGNet exhibits the best comprehensive performance according to the eight standard accuracy evaluation metrics, achieving the best performance in the $S_{\alpha }$, $F_{\beta }^{w}$, meanDic and meanIOU metrics. The meanSpe MAGNet is essentially equal to that of SINet-V2. MAGNet does not optimize this metric because it can extract the features of camouflaged objects, which readily results in a certain number of false positives. From the perspective of detection speed, the fastest methods are the lightweight algorithms, SANet and F3Net. The main innovation of these two algorithms is to increase the detection speed and reduce the computational complexity and parameters, which inevitably affects segmentation accuracy. As seen from the table, these two algorithms are the networks with the lowest segmentation accuracy in the corresponding years. It is noteworthy that the MAGNet has the highest FPS among the existing COD algorithms (>30FPS means real-time), and the GFLOPs of MAGNet rank third among all algorithms, surpassing the lightweight algorithm F3Net. Taken together, MAGNet can segment camouflage targets accurately in real-time.

**Figure 9:**
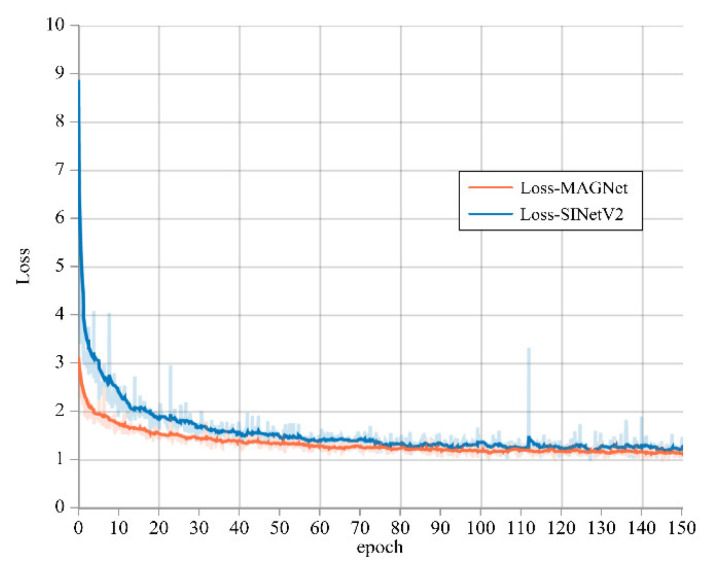
Loss value curves.

Figure 9 shows the training loss value curves of MAGNet and the optimal COD detection algorithm SINet-V2 [68]. From the figure, we can see that the loss value of MAGNet decreases faster, leveling off at 20 epochs and the final loss value is lower.

It can be seen from the table that the FPS and computational complexity of non-COD methods are generally better than that of COD methods. This is because the existing COD models based on deep learning in the pursuit of higher accuracy rates are often complex in terms of design principles and network structures. Therefore, we compared the computational complexity of all COD methods. As shown in Table 3, MAGNet’s FPS and FLOPs are ahead of other networks, and the number of parameters is essentially the same as SINet-V2. This is attributed to the clear and efficient network structure of MAGNet, which makes the model more lightweight and faster in segmentation.

#### 4.2.2. Qualitative Comparisons

**Figure 10:**
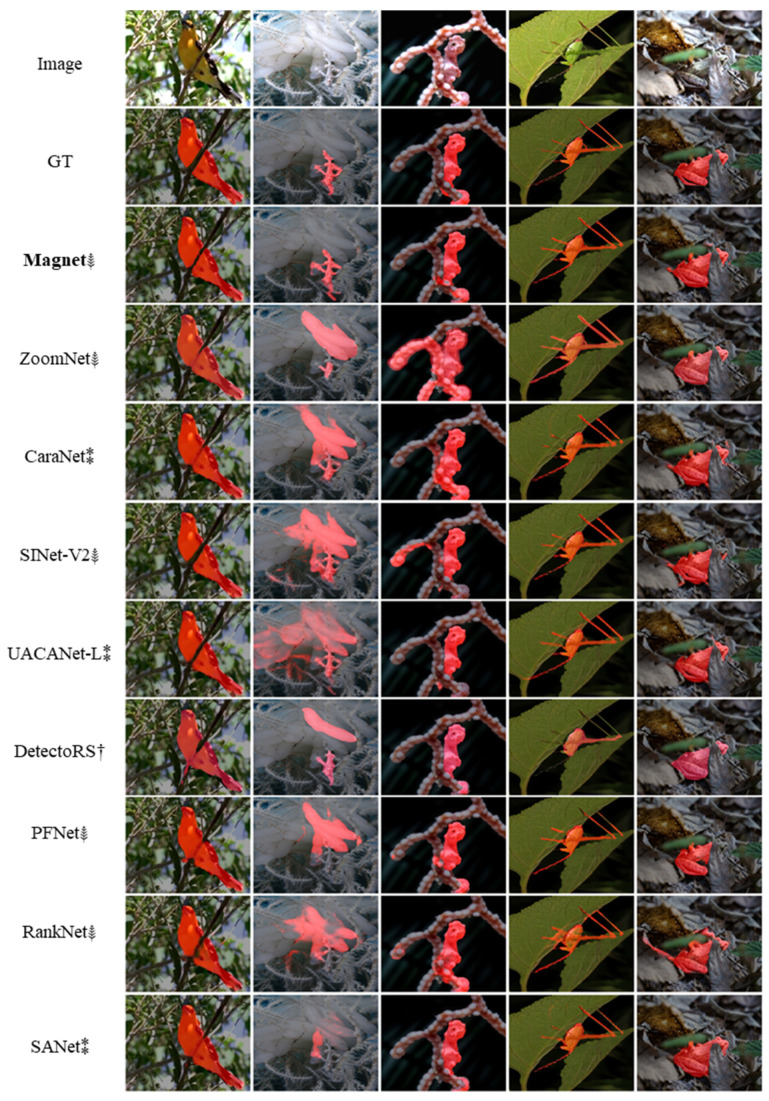
Visualization results for all algorithms on public datasets.(†: generic object detection methods, ⁑: medical image segmentation method, ⸙: COD method, bold: our method.)

As shown in Table 2, the algorithms from 2021 onwards perform better in COD. Figure 10 shows the visualization results of all algorithms since 2021. It can be observed that MAGNet can more accurately segment camouflaged targets. The EMM can better identify small targets hidden in complex backgrounds by magnifying the receptive field and fusing multi-scale features, as the hidden GhostPipefish in the second column, the MAGNet achieves the lowest missed segmentation. In contrast, most non-COD algorithms (e.g., UACANet-L, DetectoRS) tend to be more effective in salient regions of the image, and thus, do not apply to COD. AFM can acquire more important information in channels and space by simulating the human visual attention mechanism to accurately segment the details of camouflaged objects. As observed in the fifth column, MAGNet can better segment the frog’s obscured head. Using the weighted key point area, perception-loss function causes the model to focus more on the regions near the key points of a camouflaged object. As shown in the first column and the third column, MAGNet can achieve the lowest segmentation false positive rate.

### 4.3. Ablation Experiment

We conducted ablation experiments to verify the effectiveness of two specific modules designed for COD: the EMM and AFM.

#### 4.3.1. Quantitative Comparison

The results of the MAGNet ablation experiments are comprehensively reported in Table 4. Adding the two modules alone improves model performance significantly. Adding AFM optimizes meanSen due to the effect of the attention mechanism of the model, which reduces the probability of missed detection. The addition of the EMM optimizes meanSpe since the model’s receptive field magnifying mechanism works to reduce the model’s false positive probability. We also compare the results with the two key modules connected in series and parallel, ultimately finding that the parallel structure better maximizes the effects of both modules.

#### 4.3.2. Qualitative Comparisons

**Figure 11:**
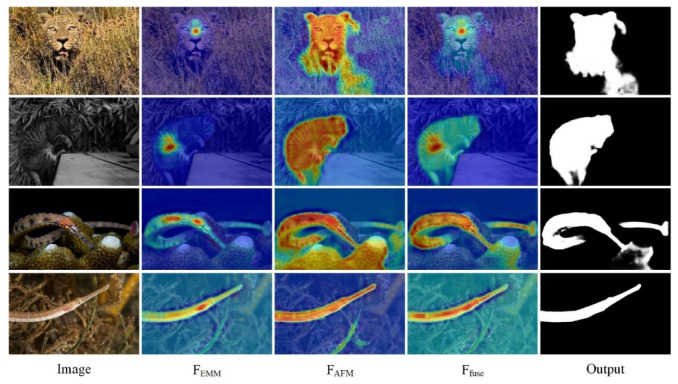
Visualization of MAGNet feature maps. (F_EMM_: output by the EMM, F_AFM_: output by the AFM, F_fuse_: final fused camouflaged object map).

We visualize the feature maps output by the EMM and AFM and compare them with the final fused camouflaged object map. The results are shown in Figure 11. The feature map output by the EMM proves that this module focuses more on the center of a camouflaged object, while the AFM can retain more important information about the target. The fused output camouflage feature map combines the advantages of both modules. The center of the camouflaged object is used as a key point to precisely find important information in the vicinity of the point, and thus, improving the accuracy of segmentation.

### 4.4. Comparison Experiment of Loss Function Parameter Settings

**Figure 12:**
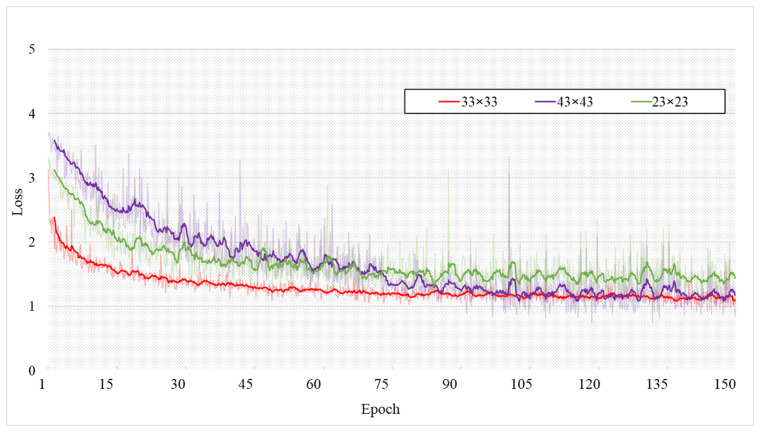
Decline curve of Loss value (solid line is the smoothed decline curve).

In Section 3.4, we detail the weighted, key-point-area perception loss. In Equation (10), h and w are the sizes of the regions around the pixel points in the GT map. We discuss the rules for the selection of *h* and *w* from a theoretical perspective, i.e., the following points need to be satisfied: (1) *h* and *w* should not be smaller than the maximum perceptual field of 32 × 32 for a single pixel; (2) should be as small as possible; and (3) should be set to an odd number. In this section, we selected 23 × 23, 33 × 33, and 43 × 43 for comparison experiments. Figure 12 shows the decreasing curve of the Loss value. From the figure, we can see that the decrease in the training loss value is not significant when set to 23 × 23 because the area involved in the calculation is smaller than the maximum perceptual field. When set to 43 × 43, the final loss value is similar to that when set to 33 × 33, but the area involved in the calculation is too large, resulting in a slight decrease in the loss value and a significant final fluctuation. Table 5 shows the quantitative evaluation of each group of experiments, and the evaluation results are not that different when set to 43 × 43. Still, it takes a longer time to train an epoch.

Therefore, experiments prove that when *h* and *w* are set to 33 × 33, they are more conducive to efficient training and can achieve the best performance.

### 4.5. Comparison of the In-House Military Camouflaged Object Dataset

**Figure 13:**
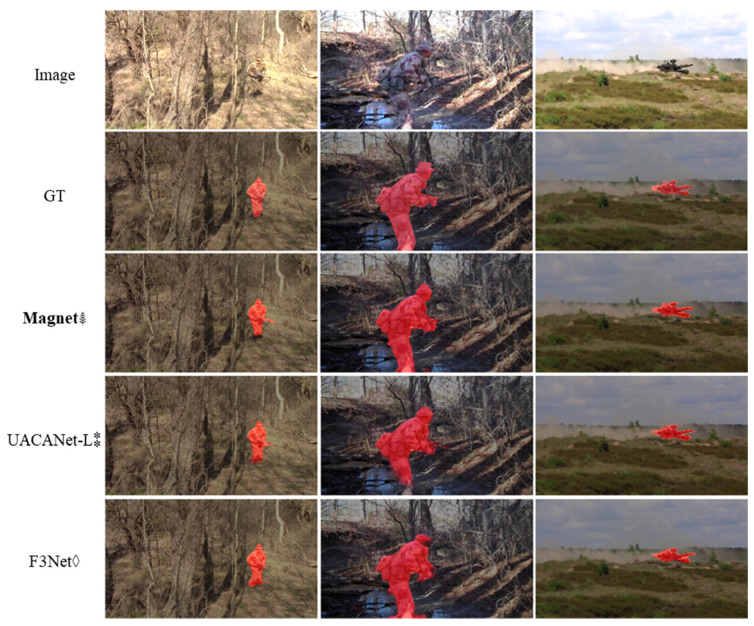
Visualization results on the in-house military camouflaged object dataset. (⁑: medical image segmentation method, ◊: saliency object detection method, ⸙: COD method, bold: our method).

Table 6 shows the experimental comparison results of the MAGNet method proposed in this paper and other methods on the military camouflaged object dataset built in-house. As seen in Table 6, MAGNet reaches the optimum in seven metrics and has the best comprehensive segmentation ability; in particular, the meanSen is improved by 6.4% compared with the next-best method UACANet-L, which means that the MAGNet model has the lowest missing detection rate. Since each image contains camouflaged objects, the meanSpe of each model is relatively high, while that of MAGNet is still 1% higher, which means that MAGNet simultaneously has the lowest false positive rate. The balance of the missed detection rate and the false positive rate is a testament to the stability of the network model and is particularly important in practical military applications. Figure 13 shows the results of the comparison experiments in this subsection on the in-house-built military camouflaged object dataset. We selected the two algorithms with the best overall performance besides MAGNet for comparison. We found that our MAGNet is better at extracting details (e.g., it can better segment the gun in the soldier’s hand, as shown in the first column) and has fewer missed regions and false alarm regions (e.g., the second and third columns).

### 4.6. Discussion

**Figure 14:**
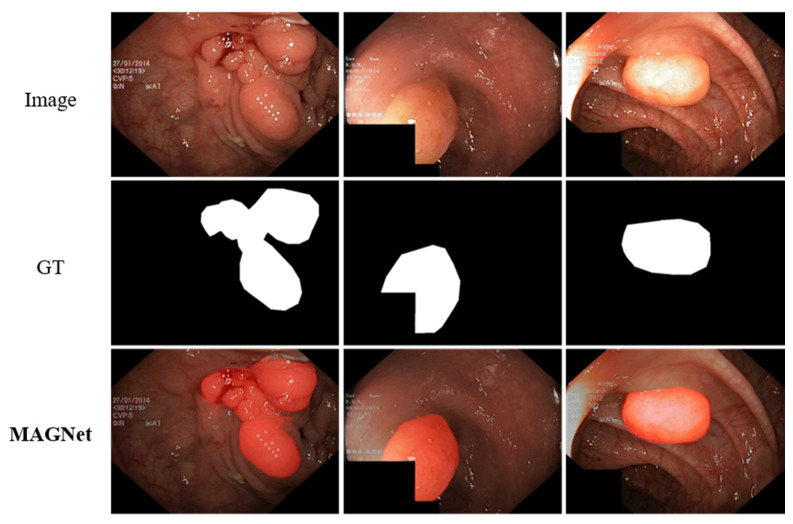
Visualization of detection results on the Kvasir-SEG polyp dataset.

From the comparison with the latest methods in Section 4.2, we find that the results of several saliency object detection algorithms are unsatisfactory, which proves that it is not reasonable to apply saliency object detection algorithms to the detection of camouflaged objects. COD methods and medical image segmentation methods accounted for 96% (23/24) of the top three values of the eight metrics. The results show that medical image segmentation methods can achieve better results in camouflaged object segmentation tasks because some medical image datasets (e.g., polyp datasets) have properties similar to those of camouflaged objects, i.e., inconspicuous edges and high integration with the surrounding environment [70,71,72]. Therefore, COD has a high potential application in the medical field. Figure 14 shows the visualization results of MAGNET applied to polyp detection, where the dataset used for the experiment is the Kvasir-SEG polyp dataset [70]. Table 7 shows the experimental comparison between the MAGNet method proposed in this paper and other medical image segmentation methods in the Kvasir-SEG polyp dataset. Here, we follow the experimental setup used in the literature [5]. The quantitative results of other algorithms are used from the original paper. It is worth noting that although MAGNet is not specifically designed for polyp detection; its performance is close to that of the best polyp-detection networks (where it achieves sub-optimal performance) and where it is evident that MAGNet has high potential for applications. When using migration learning coupled with model optimization for MAGNet, quantitative evaluation may be an even better option.

**Figure 15:**
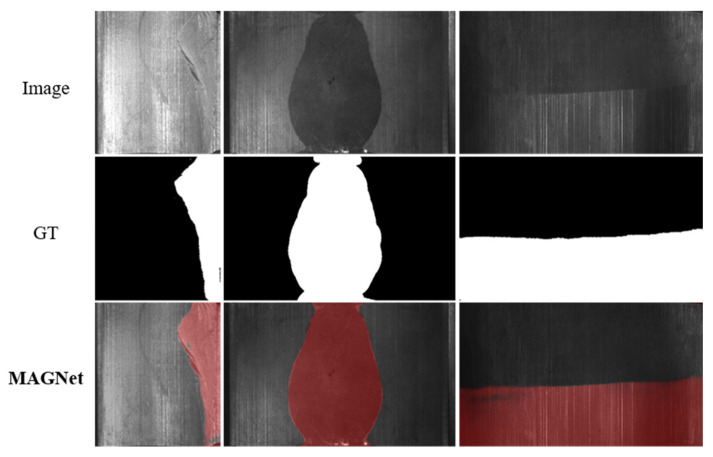
Visualization of the detection results on the magnetic tile defect dataset.

**Figure 16:**
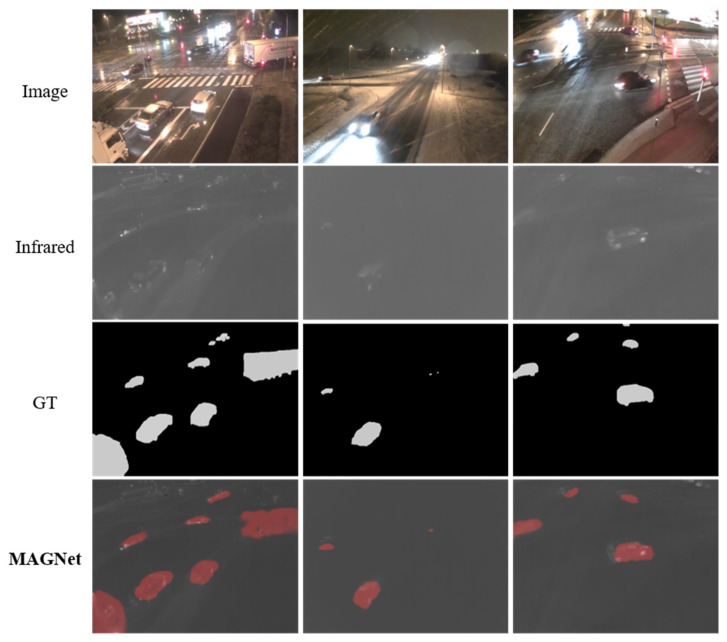
Visualization of detection results on the AAU-RainSnow dataset.

In addition, we explore other extended applications similar to COD. Figure 15 shows the visualization results applied for defect detection in industry, where the used dataset is the magnetic tile defect dataset [73]. Figure 16 shows the visualization results from applying MAGNET to infrared vehicle detection in rain and fog at night with the AAU-RainSnow dataset [74]. In these applications, similar to camouflaged objects, the object to be detected exhibits a high degree of fusion with the background, so the detection of camouflaged objects can be extended to similar applications.

## 5. Conclusions

This paper is dedicated to achieving more accurate detection of camouflaged objects. By simulating the search function of a magnifier, we propose a new network based on the observed effect of a magnifier named MAGNet. We designed two bionic modules that can be processed in parallel and presented a more applicable weighted key-point- area perception loss that allows the network to exploit important information about an object further, and thus, achieving an accurate search for camouflaged objects. The results demonstrate the accuracy advantages of MAGNet for COD through quantitative and qualitative evaluation of challenging public datasets and an in-house-built dataset. MAGNet also offers lower computational complexity and faster segmentation than other COD methods. Additionally, MAGNet has potential value for applications in other fields (e.g., medical image segmentation, nighttime vehicle detection, and industrial defect detection). In the future, we will continue to explore the accurate recognition of low-detectability objects.

## Figures and Tables

**Table 1:** Military camouflaged object dataset overview.

Categories	Descriptions	Quantities
Disguised persons	The woods in spring	800
	The woods in summer	900
	The woods in autumn	400
	The woods in winter	500
Disguised tanks	Complex environments	100
Total		2700

**Table 2:** The comparison results of MAGNet and 19 algorithms on public datasets. (†: generic ob-ject detection methods, ⁑: medical image segmentation method, ◊: saliency object detection method, ⸙: COD method, bold: our method. The top three performances are highlighted in red, blue, and green).

Methods	Pub. ‘Year	$S_{\alpha }$	$F_{\beta }^{w}$	MAE	$E_{\phi }^{ad}$	meanDic	meanIoU	meanSen	meanSpe	FPS	GFLOPs	Params (M)
UNet++ ⁑	DLMIA ‘17	0.678	0.491	0.067	0.763	0.529	0.416	0.553	0.859	60.29	106.74	24.89
MaskRCNN †	ICCV ‘17	0.756	0.643	0.042	0.790	0.625	0.534	0.653	0.803	26.90	75.82	43.75
BASNet ◊	CVPR ‘19	0.663	0.439	0.097	0.732	0.490	0.381	0.611	0.865	9.36	481.14	87.06
SCRN ◊	ICCV ‘19	0.791	0.583	0.052	0.799	0.640	0.529	0.676	0.926	35.27	30.32	25.22
HarDNet ⁑	ICCV ‘19	0.785	0.651	0.043	0.874	0.676	0.575	0.690	0.930	61.51	22.80	17.42
HTC †	CVPR ‘19	0.738	0.611	0.041	0.741	0.576	0.501	0.596	0.710	9.20	188.84	79.73
F3Net ◊	AAAI ‘20	0.781	0.636	0.049	0.851	0.675	0.565	0.709	0.940	62.12	32.86	25.54
PraNet ⁑	MICCAI ‘20	0.799	0.665	0.045	0.866	0.700	0.595	0.737	0.939	45.83	26.15	32.58
GCPANet ◊	AAAI ‘20	0.800	0.646	0.042	0.851	0.674	0.573	0.691	0.934	9.36	131.40	67.06
SINet-V1 ⸙	CVPR ‘20	0.806	0.684	0.039	0.883	0.714	0.608	0.737	0.948	37.64	38.76	48.95
Swin-S †	ICCV ‘20	0.780	0.681	0.040	0.840	0.676	0.580	0.712	0.873	14.30	89.82	68.69
SANet ⁑	MICCAI ‘21	0.791	0.659	0.046	0.862	0.702	0.593	0.766	0.938	69.09	22.56	23.90
RankNet ⸙	CVPR ‘21	0.799	0.661	0.043	0.860	0.696	0.588	0.723	0.947	29.51	66.63	50.94
PFNet ⸙	CVPR ‘21	0.805	0.683	0.040	0.882	0.714	0.607	0.737	0.951	33.74	53.24	46.50
DetectoRS †	CVPR ‘21	0.804	0.725	0.039	0.851	0.712	0.624	0.739	0.861	5.50	188.36	134.00
UACANet-L ⁑	ACMMM ‘21	0.816	0.724	0.034	0.901	0.745	0.646	0.763	0.945	23.19	119.05	69.6
SINet-V2 ⸙	TPAMI ‘21	0.822	0.700	0.038	0.883	0.735	0.627	0.767	0.955	52.20	24.48	26.98
CaraNet ⁑	MIIP ‘22	0.815	0.679	0.044	0.862	0.722	0.618	0.789	0.937	31.88	43.30	46.63
ZoomNet ⸙	CVPR ‘22	0.818	0.703	0.037	0.875	0.721	0.625	0.716	0.941	12.06	203.50	32.38
**MAGNet ⸙**	**Ours**	**0.829**	**0.727**	**0.034**	**0.901**	**0.757**	**0.656**	**0.789**	**0.954**	**56.91**	**24.36**	**27.12**

**Table 3:** Computational complexity comparison results of MAGNet and other COD methods (based on https://github.com/lartpang/MethodsCmp (accessed on 1 November 2022) [69]. ⸙: COD method, bold: our method. The top three performances are highlighted in red, blue, and green.).

Methods	Pub. ‘Year	FPS	FLOPs (G)	Params (M)
SINet-V1 ⸙	CVPR ‘20	37.64	38.76	48.95
RankNet ⸙	CVPR ‘21	29.51	66.63	50.94
PFNet ⸙	CVPR ‘21	33.74	53.24	46.50
SINet-V2 ⸙	TPAMI ‘21	52.20	24.48	26.98
ZoomNet ⸙	CVPR ‘22	12.06	203.50	32.38
**MAGNet ⸙**	**Ours**	**56.91**	**24.36**	**27.12**

**Table 4:** MAGNet ablation experiment results.(✓ indicates that the module is used. The top three performances are highlighted in red, blue, and green).

Baseline	With AFM	With EMM	In Series	In Parallel	$S_{\alpha }$	$F_{\beta }^{w}$	MAE	$E_{\phi }^{ad}$	meanDic	meanIoU	meanSen	meanSpe
✓					0.663	0.315	0.151	0.711	0.522	0.399	0.761	0.826
✓	✓				0.675	0.308	0.163	0.843	0.616	0.509	0.824	0.812
✓		✓			0.825	0.715	0.035	0.900	0.742	0.638	0.755	0.956
✓	✓	✓	✓		0.827	0.723	0.034	0.902	0.753	0.652	0.785	0.949
✓	✓	✓		✓	0.829	0.727	0.034	0.901	0.757	0.656	0.789	0.954

**Table 5:** Comparison results with different parameter settings. (The last column is the time required to train an epoch. The top performance are highlighted in red).

Settings	$S_{\alpha }$	$F_{\beta }^{w}$	MAE	$E_{\phi }^{ad}$	meanDic	meanIoU	meanSen	meanSpe	Time/s
23 × 23	0.809	0.644	0.046	0.847	0.719	0.610	0.787	0.946	137.2
43 × 43	0.824	0.723	0.034	0.903	0.746	0.648	0.760	0.952	146.9
33 × 33	0.829	0.727	0.034	0.901	0.757	0.656	0.789	0.954	142

**Table 6:** Comparison results on the in-house military camouflaged object dataset. (†: generic object detection methods, ⁑: medical image segmentation method, ◊: saliency object detection method, ⸙: COD method, bold: our method. The top three performances are highlighted in red, blue, and green.)

Methods	Pub. ‘Year	$S_{\alpha }$	$F_{\beta }^{w}$	MAE	$E_{\phi }^{ad}$	meanDic	meanIoU	meanSen	meanSpe
UNet++ ⁑	DLMIA ‘17	0.717	0.594	0.009	0.736	0.513	0.421	0.471	0.747
MaskRCNN †	ICCV ‘17	0.825	0.762	0.008	0.856	0.695	0.543	0.746	0.874
BASNet ◊	CVPR ‘19	0.865	0.757	0.008	0.928	0.763	0.666	0.758	0.950
SCRN ◊	ICCV ‘19	0.847	0.603	0.010	0.677	0.687	0.575	0.726	0.955
HarDNet ⁑	ICCV ‘19	0.876	0.784	0.005	0.953	0.795	0.695	0.806	0.967
HTC †	CVPR ‘19	0.848	0.766	0.006	0.824	0.753	0.504	0.764	0.858
F3Net ◊	AAAI ‘20	0.889	0.798	0.005	0.944	0.816	0.716	0.846	0.972
PraNet ⁑	MICCAI ‘20	0.887	0.781	0.006	0.915	0.802	0.696	0.834	0.977
GCPANet ◊	AAAI ‘20	0.874	0.721	0.006	0.821	0.733	0.623	0.714	0.971
SINet-V1 ⸙	CVPR ‘20	0.876	0.800	0.005	0.965	0.810	0.706	0.842	0.977
Swin-S †	ICCV ‘20	0.858	0.710	0.008	0.834	0.741	0.635	0.837	0.951
SANet ⁑	MICCAI ‘21	0.804	0.647	0.010	0.853	0.673	0.563	0.720	0.917
RankNet ⸙	CVPR ‘21	0.847	0.693	0.008	0.825	0.737	0.622	0.840	0.960
PFNet ⸙	CVPR ‘21	0.873	0.771	0.006	0.941	0.785	0.682	0.804	0.965
DetectoRS †	CVPR ‘21	0.863	0.784	0.007	0.917	0.803	0.698	0.826	0.965
UACANet-L ⁑	ACM MM ‘21	0.880	0.823	0.004	0.963	0.817	0.715	0.853	0.979
SINet-V2 ⸙	TPAMI ‘21	0.884	0.788	0.004	0.926	0.806	0.699	0.843	0.982
CaraNet ⁑	MIIP ‘22	0.865	0.729	0.006	0.873	0.763	0.654	0.832	0.964
ZoomNet ⸙	CVPR ‘22	0.881	0.798	0.005	0.888	0.783	0.685	0.784	0.965
**MAGNet ⸙**	**Ours**	**0.924**	**0.864**	**0.003**	**0.946**	**0.868**	**0.779**	**0.917**	**0.992**

**Table 7:** Comparison results on the Kvasir-SEG polyp dataset. (⁑: medical image segmentation method, ⸙: COD method, bold: our method. The top three performances are highlighted in red, blue, and green.)

Methods	Pub. ‘Year	meanDic	MAE	$S_{\alpha }$	$E_{\phi }^{ad}$	meanIoU
UNet++ ⁑	DLMIA ‘17	0.821	0.048	0.862	0.910	/
HarDNet ⁑	ICCV ‘19	0.912	0.025	0.923	0.958	0.857
PraNet ⁑	MICCAI ‘20	0.898	0.030	0.915	0.948	0.849
UACANet-L ⁑	ACM MM ‘21	0.912	0.025	0.917	0.958	0.862
CaraNet ⁑	MIIP ‘22	0.918	0.023	0.929	0.968	0.865
**MAGNet ⸙**	**Ours**	**0.890**	**0.033**	**0.912**	**0.960**	**0.830**

## Data Availability

Data related to the current study are available from the corresponding author upon reasonable request. The codes used during the study are available from the corresponding author upon request. Additionally, some of the codes and results of this paper can be found at: https://github.com/jiangxinhao2020/Magnet_eval (accessed on 1 December 2022).
